# Presence probability of *Hemiscorpius lepturus* Peters, 1861 using maximum entropy approach in the western areas of Zagros Mountains, Iran

**DOI:** 10.14202/vetworld.2020.296-303

**Published:** 2020-02-17

**Authors:** Ahmad Ali Hanafi-Bojd, Mona Sharififard, Elham Jahanifard, Shahrokh Navidpour, Babak Vazirianzadeh

**Affiliations:** 1 Department of Medical Entomology and Vector Control, School of Public Health, Tehran University of Medical Sciences, Tehran, Iran; 2 Social Determinants of Health Research Center, Ahvaz Jundishapur University of Medical Sciences, Ahvaz, Iran; 3 Department of Medical Entomology and Vector Control, School of Public Health, Ahvaz Jundishapur University of Medical Sciences, Ahvaz, Iran; 4 Razi Reference Laboratory of Scorpion Research, Razi Vaccine and Serum Research Institute, Agricultural Research Education and Extension Organization, Karaj, Iran

**Keywords:** ecological niches, *Hemiscorpius lepturus*, Iran, MaxEnt, Zagros mountains

## Abstract

**Aim:**

The purpose of this research was to use environmental variables for predicting the probability of *Hemiscorpius lepturus* existence in the provinces where situated in the west of the Zagros Mountains.

**Materials and Methods:**

In this study, 64 occurrence records of the *H. lepturus* were extracted from the published documents available in electronic databases. MaxEnt model was used for predicting the ecological niches of this species. Normalized difference vegetation index (NDVI) and 19 climatic variables were used as the environmental variables affecting the distribution of this scorpion. The Jackknife test in the model was used to indicate the importance of variables to predict the probability of the presence of the studied species. The logistic threshold that was evaluated using a logistic regression algorithm showed the converting of the probability model into a binary model. The model was evaluated byarea under the curve (AUC). The probability presence map of this scorpion was then prepared in ArcGIS 10.5 Software.

**Results:**

The results of the analysis showed that the most important environmental factor on the distribution of *H. lepturus* was the maximum temperature of the warmest month (Bio5) with a contribution rate of 43% and permutation importance of 8%. The Jackknife test revealed that NDVI did not gain any value when it used independently in the model. The logistic threshold was reported 0.255 for the maximum test sensitivity plus specificity. The AUC of the model was 0.7698, shows an acceptable value for model validity. Overall the hot spots for this toxic scorpion seem to be in Khuzestan, Lorestan, and Ilam Provinces of the studied area.

**Conclusion:**

Regarding our findings, MaxEnt algorithm, in combination with geographic information system contributed to revealing the effects of environmental variables on the probability of *H. lepturus* presence in the west of Zagros Mountains. These visualized maps as a warning alarm can be helpful to policymakers for managing, controlling, and monitoring the scorpionism in high-risk areas.

## Introduction

Scorpions are important medical arthropods that belong to the class of *Arachnida* and *scorpionidae* order that are active at night and are mainly found in hot and dry areas [1]. These animals are not living fossils due to various adaptations, including behavioral, physiological, and ecological which ensure their success during 450 million years ago [2]. The arthropods have a venomous sting and almost 1500 species of scorpions are known worldwide, about 30 of which are potentially dangerous to humans [3]. Nowadays, scorpions are found in terrestrial habitat and Iran is a rich region for many species of dangerous scorpions due to climatic conditions [1]. There are three families of *Scorpionidae*, Hemiscorpiidae, and *Buthidae* in Iran that 86% of species is related to *Buthidae* [4]. Scorpion sting is one of the major health problems in Iran that has caused many deaths in the south and southwestern part of the country [5]. According to the incommunicable Disease Management Center of Ministry of Health and Medical Education, more than 50,000 stings are reported in the country. Although the most scorpionism and deaths occur in Khuzestan Province, it is reporting from other parts of the country as well [1]. The average of the incidence of scorpionism is about 59.5/100,000 population in Iran. Most cases of scorpion stings happened in Khuzestan Province with 541/100,000 population. Moreover, Hormozgan, Bushehr, and Ilam provinces with 15.9, 127, and 123/100,000 are in the next position, respectively [6]. The incidence and severity of clinical signs of scorpion sting depend on the species of scorpion, the physiological conditions of the patient, the number of stings, the amount of toxin injected, the age, weight, and health of the patient. Clinical effects can range from simple localized pain to severe systemic reactions that sometimes lead to death [7].

An epidemiological survey of envenomation showed that there are at least seven medically important scorpion species in Iran. One of them is *Hemiscorpius lepturus* (Gadim) that belongs to the Hemiscorpiidae family [6]. It worth noting that one genus (*Hemiscorpius*) and seven species (*Hemiscorpius acanthocercus*, *Hemiscorpius enischnochela*, *Hemiscorpius gaillardia*, *Hemiscorpius lepturus*, *Hemiscorpius persicus*, *Hemiscorpius kashkayi*, and *Hemiscorpius shahii*) of this family reported from Iran [8, 9]. The family Hemiscorpiidae is distributed in East Africa, the Arabian Peninsula, Iraq, and Iran [10]. Moreover, *H. lepturus* as a non-digger scorpion distributed in 15 of 31 provinces in the country such as Khuzestan, Hormozgan, Bushehr, Kerman, Fars, Kohgiluyeh and Boyer Ahmad, Chaharmahal and Bakhtiari, Ilam, and Lorestan [8,11-21]. The color of this scorpion is light yellow to brown. While, moveable and fixed fingers of pedipalps are reddish. Sexual dimorphism was observed in this species, such that males have significantly longer metasoma than females [22]. The scorpion has a longer carapace than wide with stout and bulky pedipalps. The vesicle of telson is ovoid, globular, and bulky with fine and thin stings. The size of males can vary greatly from 52 mm up to 85 mm body length; females display less variation in size. Furthermore, 8-11 teeth and 14-16 teeth exist in pectines of females and males, respectively [23]. The venom of *H. lepturus* is composed of cytotoxins and hemotoxins. And also, it had a greater effect on erythrocyte hemolysis than other important species, *Androctonus crassicauda*, in the area. Its symptoms can include hematuria, acute and secondary renal failure, skin rashes, blistering, and necrosis of scorpion sting and surrounding tissues and very little pain such as itching or bee stings [8]. The severe hemoglobinuria observed in 95% of *H. lepturus* victims. Previous studies showed that the highest fatality rate in Khuzestan Province was related to envenomation by *H. lepturus*. This scorpion is responsible for 10-25% of the scorpion sting in the warm seasons and throughout the winter. Moreover, 24.9% of total scorpionism in Khuzestan Province is due to Gadim [24]. Children are at risk group of *H. lepturus* sting with high mortality among them [25]. The emergency injection of antivenom is essential in *H. lepturus* envenomation due to hemolysis and, consequently, acute renal failure in the first 24 h after scorpion sting [26]. Intramuscular injection of antivenoms is ineffective in neutralizing the action of venom [27]. While the intramuscular injection route of antivenom is useful, only under 2 h following *H. lepturus* sting [28].

Species distribution models are analytical or statistical algorithms that can predict the actual and potential species distribution by linking among field observations and environmental factors [29]. One of the methods for determining the habitat is the use of predictive models of dispersal that their base is quantifying the relationship between species and different environmental variables [30]. Models based on the concept of ecological niche modeling provide good information on possible species distribution when there is insufficient data and also can be used in species conservation planning [29]. One of these methods is the maximum entropy or MaxEnt, which is used to represent species distribution [31]. The MaxEnt model is a machine learning based on the maximum disorder that is used to predict the presence when data are not available in the region. This method estimates the probability of the presence of a species based on the constraints obtained from existing data [32]. MaxEnt is a program for modeling the species distribution of the collected samples. Furthermore, this method is used for various aims including predicting the potential distribution of species, phyloclimatic studies, understanding environmental and ecological correlates of species occurrences [33]. Ecological and climatic variables in analyses based geographic information system (GIS) reported as determining the species boundaries [34]. GIS in combination with ecological niche modeling was applied to preparing the risk maps and predict the distribution of some medically important insects and arthropods [29, 35-38].

The purpose of this study was to use environmental factors in combination with the MaxEnt model for predicting the probability of *H. lepturus* presence as the dependent variable in the provinces in the west of the Zagros Mountains in Iran. The model prediction ability was evaluated by the AUC value. Furthermore, we can determine the contribution value of each environmental variable in the distribution of the scorpion in study areas. The present study is able to complete zoo-geographical data to design the management strategies based on ecological niche modeling and to prepare the visualize map for monitoring the scorpionism in high-risk regions.

## Materials and Methods

### Ethical approval

All articles that used in the present study were referenced in the paper. Furthermore, this study was approved by the Ethical Committee on December 4, 2016, at Ahvaz Jundishapur University of Medical Sciences, Iran (No. IR.AJUMS.REC.1395.538).

### Study area

Iran, a country in the southwest of Asia and the Middle East, with 32.4279° N and 53.6880° E covers an area of 1,648,195 km^2^. According to data from Statistical Center of Iran, it ranks 18^th^ in the World with 79,926,270 human population. It is bordered by Azerbaijan, Armenia, and Turkmenistan to the north, Afghanistan, and Pakistan to the east, and Turkey and Iraq to the west. It also limited to the Caspian Sea from the north, and the Persian Gulf and Oman Sea to the south, which are the first two major oil and gas extraction sites in the world.

**Figure-1:**
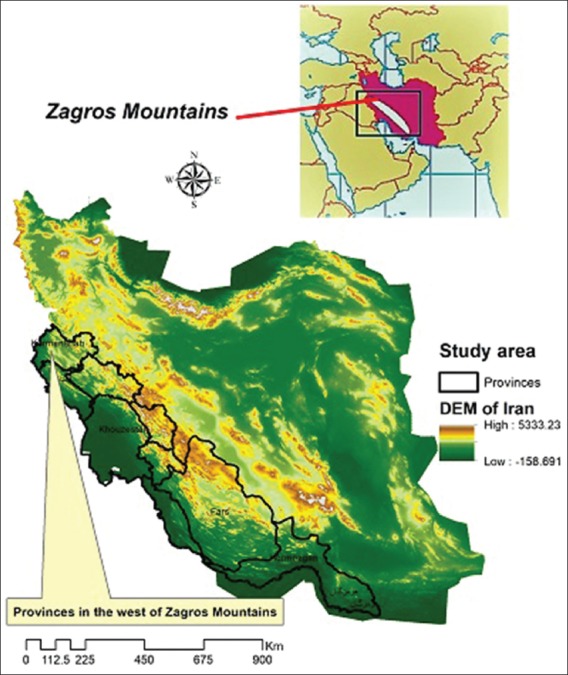
Study areas in the west of Zagros Mountains [Map prepared by EJ].

The Zagros Mountains are a long mountain range in Iran, Iraq, and Southeast Turkey. This mountain range has a total length of 1600 km. The Zagros mountain range begins in Northwest Iran and roughly follows Iran’s western border, while covering much of Southeast Turkey and Northeast Iraq. Moreover, the provinces located west of Zagros Mountains in Iran are Kermanshah, Chaharmahal andBakhtiari, Bushehr, Fars, Khouzestan, Ilam, Lorestan, Kohgiluyeh dnaBoyer-Ahmad, and Hormozgan (Figure-1).

### Data collection

The occurrence record of *H. lepturus* was extracted from the published documents by searching on databases such as Google Scholar, PubMed, Scopus, Iran Medex, Elsevier, and Scientific Information Database. Fauna, biodiversity, scorpion, species composition, and Iran were used as keywords for the search strategy. Researches that had been down in Kermanshah, Chaharmahal and Bakhtiari, Bushehr, Fars, Khuzestan, Ilam, Lorestan, Kohgiluyeh and Boyer Ahmad, and Hormozgan Provinces and collected *H. lepturus* scorpion were included in the study. The exclusion criteria were regarded as other species except for *H. lepturus*. Moreover, latitude and longitude of sampling locations were used for creating the species databank. All data designed in Microsoft Excel and its table added to ArcGIS 10.5. Then, shapefile of *H. lepturu* s prepared in ArcMap application and it was used for showing geographical distribution and modeling of the presence chance of *H. lepturus*.

### MaxEnt modeling

MaxEnt is suitable for modeling species’ geographic distributions with available information, including climatic data and land cover [32]. Environmental variables include 19 items (Bio1: Annual Mean Temperature (°C); Bio2: Mean diurnal range (mean of monthly [max temp–min temp]) (°C); Bio3: Isothermality (Bio2/Bio7) (×100); Bio4: Temperature seasonality (standard deviation × 100); Bio5: Max temperature of warmest month (°C); Bio6: Min temperature of coldest month (°C); Bio7: Temperature annual range (Bio5–Bio6) temperature of wettest quarter (°C); Bio8: Mean temperature of wettest quarter (°C); Bio9: Mean temperature of driest quarter (°C); Bio10: Mean temperature of warmest quarter (°C); Bio11: Mean temperature of coldest quarter (°C); Bio12: Annual precipitation (mm); Bio13: Precipitation of wettest month (mm); Bio14: Precipitation of driest month (mm); Bio15: Seasonal precipitation (coefficient of variation); Bio16: Precipitation of wettest quarter (mm); Bio17: Precipitation of driest quarter (mm); Bio18: Precipitation of warmest quarter (mm); and Bio19: Precipitation of coldest quarter) in raster format with spatial resolution of 1 km^2^ were downloaded from the WorldClim database (http://www.worldclim.org/current). Normalized difference vegetation index (NDVI) gained from the image of moderate resolution imaging spectroradiometer satellite in 2014. Eighty percent of the occurrence recodes of *H. lepturus* were randomly used by the software for training the model and the remaining 20% for the test.

The logistic threshold that was evaluated using a logistic regression algorithm showed the converting of the probability model into a binary model.

The Jackknife Estimation method used to indicate the association of all variables to predict species distribution. The accuracy of modeling geographic distribution of Gadim was evaluated by receiver operating characteristic (ROC) curve. The area under an ROC curve (AUC) is between maximum 1 and minimum 0 [39]. An excellent model has AUC near to the one which means it has a good measure of separability. Moreover, AUC near to zero means it has the worst measure of separability and the model is poor. The model performance regarding AUC was classified into failing, poor, fair, good, and excellent in the range of 0.5-0.6, 0.6-0.7, 0.7-0.8, 0.8-0.9, and 0.9-1, respectively [40].

## Results

The ecological niche model, MaxEnt, predicted the presence probability of *H. lepturus* in the west of Zagros Mountains. Regarding previous studies, 64 points were used for modeling this species, 48 records for training, and 16 for testing the model.

The relative contributions of all environmental variables were estimated in this model. The results of the analysis showed that the most important environmental factors in the distribution of *H*. *lepturus* were the maximum temperature of the warmest month (Bio5) contributed 43% with 8% permutation importance. Furthermore, Bio6 (min temperature of the coldest month), Bio8 (mean temperature of the wettest quarter), and Bio16 (precipitation of wettest quarter) did not contribute to MaxEnt algorithm.

**Figure-2:**
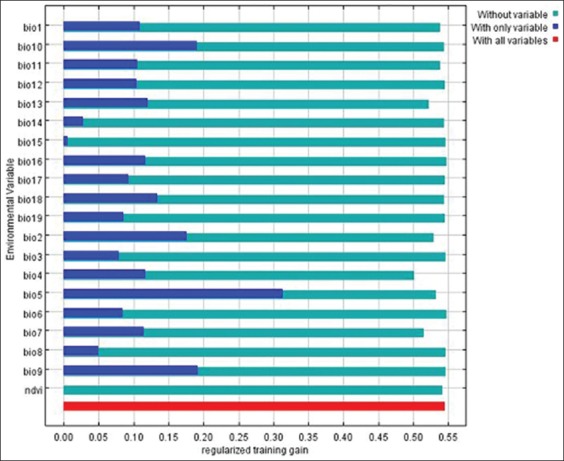
Jackknife of regularized training gain for *Hemiscorpius lepturus* in the west of Zagros Mountains in Iran.

The results of the Jackknife test related to variable important are indicated in Figure-2. The climatic variable of bio5 had the most useful information in predicting *H. lepturus*. Mean diurnal range (°C), mean temperature of the driest quarter (°C), and mean temperature of the warmest quarter (°C) had moderate value when they used independently. Temperature seasonality (°C) is an environmental variable that has the most information toward other variables. With missing bio4, the estimation of environmental variables in modeling is reduced. The NDVI did not gain any value when it used independently in the model.

**Figure-3:**
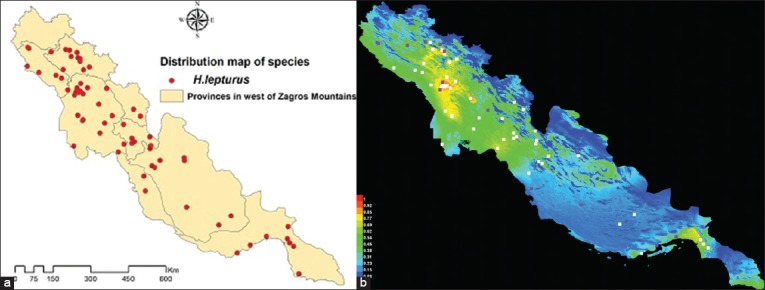
Spatial distribution (a) and prediction (b) of *Hemiscorpius lepturus* in the west of Zagros Mountains in Iran.

**Figure-4:**
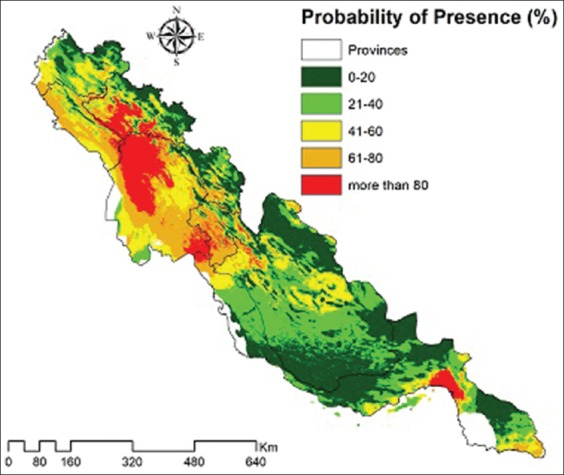
Presence probability of *Hemiscorpius lepturus* in the west of Zagros Mountains.

Geographical distribution and collecting sites of the species in study areas are indicated in Figure-3a. There was no valuable and published data on the presence of *H. lepturus* in Kermanshah Province but it is reported from other provinces in the west of Zagros Mountain. Figure-3b is a representation of the MaxEnt model for *H. lepturus* that the better predicted conditions are indicated by the warmer colors. The presence probability of the species by percentage is shown in Figure-4 in five classes (0-20, 21-40, 41-60, 61-80, and more than 80). In other words, the presence chance of *H. lepturus* increases in the last classes that are equal to the warmest color. The most probability of the scorpion existence is predicted in Khuzestan Province and followed by Lorestan and Ilam provinces.

The binomial probabilities were calculated exactly if the number of test samples was at most 25. The logistic threshold was reported 0.255 for the maximum test sensitivity plus specificity. Furthermore, thresholds resulting from MaxEnt for maximum training sensitivity plus specificity, equal training sensitivity and specificity and balance training omission, and predicted area and threshold value were calculated 0.422, 0.467, and 0.131, respectively.

**Figure-5:**
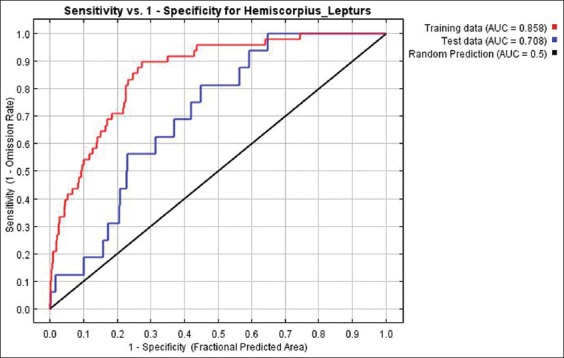
Model suitability using the AUC for training and test data.

The area under the curve (AUC) for training and test data was 0.858 and 0.708, respectively (Figure-5). When AUC is 0.7, it means there is 70% chance that the model will be able to distinguish between positive class and negative class. The model performance was in the range of 0.7-0.8 and it was evaluated fairly.

## Discussion

The majority of researches in Iran has focused on the fauna, diversity, and spatial distribution of scorpions, but the present research is able to predict this species in areas where no data are available. Regarding this study, the geographical distribution of *H. lepturus* focused especially on the provinces in the west and southwest of Iran, where the climate was hot and humid in most of them. Epidemiological studies showed that the major envenomation of *H. lepturus* happened in the southwest of the country [8]. Despite, reports of this species in Isfahan and Semnan Provinces [8], the highest distribution of this species is observed in the west of Zagros Mountains, which may be due to these mountains as a natural barrier to its geographical distribution.

The mean temperature of the wettest quarter of the year, precipitation of the coldest quarter of the year, and precipitation of the warmest quarter of the year were the most important variables that contributed to the distribution of *Mesobuthus eupeus*. Moreover, precipitation of the warmest quarter of the year as a variable had the highest contribution on *Mesobuthus phillipsii* distribution [30]. Lira *et al*. [41] showed the frequency of *Tityus pusillus* depends on environmental factors. Furthermore, another study confirmed the abundance fauna of the animal influences by response to the environmental condition [42]. Furthermore, El Hidan *et al*. [43] reported some species such as *Androctonus amoreuxi*, *Androctonus liouvillei*, *Buthus mariefranceae*, *Buthus draa*, *Buthus boumalenii*, and *Buthus bonito* adapted to Saharan bioclimate with rainfall <150 mm and hot temperature. Our result showed that the relative contributions of the environmental variable were various from the previous study and it seems that the contributing factors are specialized for every species.

Our results presented that NDVI did not gain any value in predicting the presence of the species when it used independently in the model. *Scorpio maurus* indicated a negative correlation with vegetation cover [44]. *H. lepturus* as a lithophilous species lives in laminated rocks and its morphotype is affected by the special habitat [15] which seems to indicate that this variable is not involved in the distribution of this scorpion.

The distribution of scorpion species is associated mainly with climatic factors such as temperature, precipitation, and the nature of substratum such as soil texture and hardness, the thickness, and the amount of the stones [43]. Furthermore, rainfall, relative humidity, and temperature were known as the most dominant variables in species distribution, just like that *Buthus malhommei* and *Buthus elmoutaouakili* were frequently observed in the areas with low rainfall in the range of 150 mm-350 mm and high temperature [43].

Temperature variables were a good predictor to the distribution of *Chibchea salta* by maximum entropy [45]. El Hidan *et al*. [43] found that the environment has a significant role to show the presence of *Androctonus* Genus species. Moreover, environmental factors, including topography and climate, are two important variables that can affect on envenomation cases and frequency of venomous animals. However, they found the strongest relationship between the ranges of specific environmental factors with scorpion species. Some climatic circumstances, such as precipitation of coldest and warmest quarter, influenced *Androctonus mauritanicus*. However, the mean diurnal range, slope, wettest quarter precipitation, and warmest quarter were associated with *A. liouvillei* distribution. While *A. amoreuxi* was related to the mean temperature of the driest quarter, mean diurnal range, and slope [46]. In this study, according to the MaxEnt modeling, the important climatic factor in the presence of *H. lepturus* was the maximum temperature of the warmest month. It shows that it tends to live in warm areas that such bio-climates have been provided for the living of this scorpion in south and southwest of Iran.

The area under an ROC curve was calculated 0.950±0.025, 0.988±0.006, and 0.969±0.007 for *A. amoreuxi*, *A. liouvillei*, and *A. mauritanicus*, respectively [46]. Brites-Neto and Duarte [38] reported AUC=0.7698±0.0533 for *Tityus serrulatus* and pointed to high predictive success. The results of this study presented that AUC for test data was 0.708±0.048 which was evaluated fair by Swets’ classification. The possibility map of *H. lepturus* can use as recognizing the people at risk and prevention of them from scorpion envenomation. Our result reflects that the accuracy of *H. lepturus* modeling was evaluated fairly. However, the most probability of the species presence predicted in Khuzestan Province and followed by Lorestan and Ilam, where the main habitats for the scorpion were consecutive years due to suitable climates and environment [8]. Epidemiological studies showed that *H. lepturus* was a principal cause of 10-15% of all hospital referred scorpion envenomation [8].

The strong point of this study is determining the appropriate species distribution only with presence data and recognizing absence data that are rarely available or reliable. Conclusions and interpretation of the results depend on the selected factors for analysis that can regard as weak points of the research. In our study, MaxEnt model was used to determine the distribution of *H. lepturus* by 19 environmental variables that show the limitation of our prediction to the validity of data.

## Conclusion

Regarding our findings, the MaxEnt algorithm in combination with GIS contributed to revealing the effects of environmental variables on the probability of *H. lepturus* presence in the west of Zagros Mountains. Furthermore, both tools are able to produce the risk maps of the species stings where its envenomation is a serious public health problem. Considering the importance and distribution of this species in hot areas such as Khuzestan Province, preparing the scorpion envenomation risk map is possible by adding the demographical variables and interventional activities in ArcGIS 10.5. Based on our prediction results, we suggest using the visualized maps as a warning alarm as an appropriate tool to policymakers to manage, control, and monitor the scorpion stings in high-risk areas. Furthermore, raising the awareness, knowledge, and practice of exposed people to the scorpion will decrease scorpionism.

## Authors’ Contributions

MS, SN, and BV collected data, AAH analyzed data by MaxEnt and ArcGIS. EJ prepared the article. All authors participated in editing of the manuscript. All authors read and approved the manuscript.
